# The GIST of It: A Rare Presentation of Neurofibromatosis Type I

**DOI:** 10.7759/cureus.16034

**Published:** 2021-06-29

**Authors:** Amit R Hudgi, Mohammad Azam, Muaaz Masood, Hafiz Muhammad Sharjeel Arshad, John Erikson L Yap

**Affiliations:** 1 Internal Medicine, Medical College of Georgia at Augusta University, Augusta, USA; 2 Gastroenterology and Hepatology, Medical College of Georgia at Augusta University, Augusta, USA

**Keywords:** acute gastrointestinal bleed, neurofibromatosis type 1 (nf-1), gastrointestinal stromal tumor (gist), neurofibromas, gastroenterology and endoscopy

## Abstract

Neurofibromatosis-1 (NF-1) is an autosomal dominant condition characterized by cutaneous pigmentation and tumour formation along nerves in the brain, skin, and other organs. Gastrointestinal stromal tumours (GIST) are rare mesenchymal tumours involving the gastrointestinal tract (GI) associated with NF-1. We present a case of life-threatening GI bleeding from GIST in a patient with NF-1. In NF-1 patients presenting with GI bleeding, GISTs should be part of the differential. Clinicians must have a low threshold for urgent abdominal imaging if endoscopy does not detect the source of GI bleeding.

## Introduction

Gastrointestinal (GI) bleeds are a common cause of hospital admissions [1-2]. Peptic ulcer disease remains the most common cause of GI bleeding, with less than 1% GI bleed attributed to Gastrointestinal stromal tumors (GIST) [3]. Diagnosis and management of patients with severe GI bleeding often involve endoscopy. Unfortunately, even colonoscopy is not a guarantee to find the etiology of the lower GI bleeding site. It can only identify lower GI bleeding source in 45-90% of cases [4]. Further investigation modalities include video capsule endoscopy, nuclear medicine tagged studies, and CT imaging with contrast.

This case report was previously presented as a poster at the American College of Gastroenterology 2020 Virtual Annual Meeting on October 23 - 28, 2020.

## Case presentation

**Figure 1:**
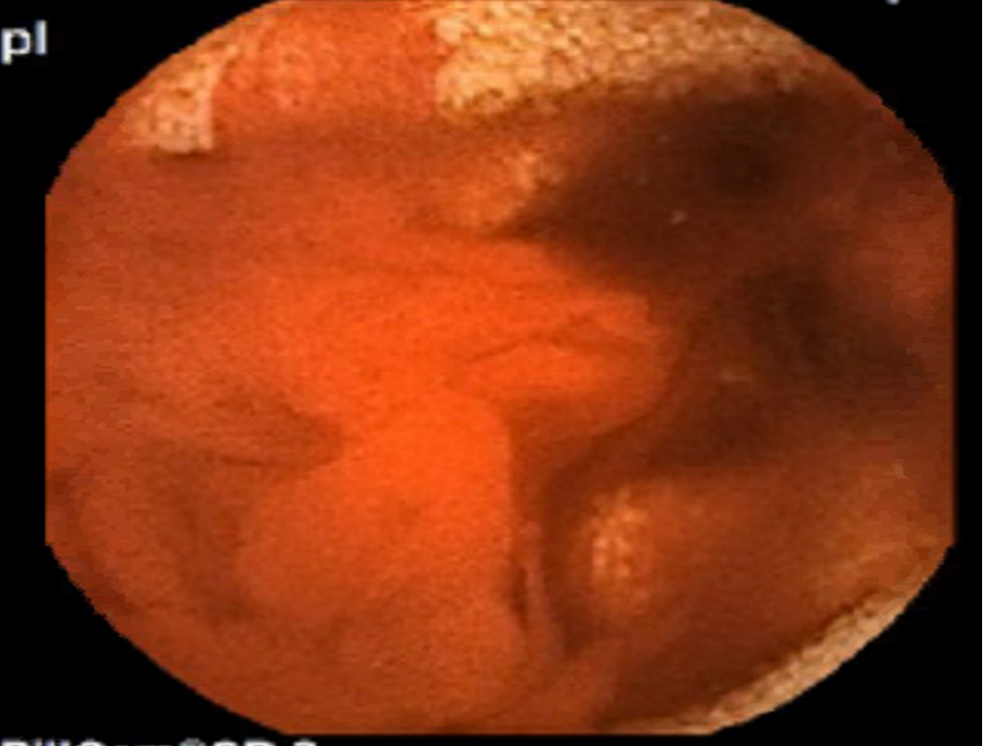
Capsule endoscopy – Brisk active bleeding lesion noted on the capsule endoscopy.

A 42-year-old female patient presented with acute onset hematochezia. She had a past medical history of NF-1 and chronic intermittent headaches with infrequent non-steroidal anti-inflammatory drugs (NSAIDs). She denied NSAID use in the last three months. She had no hematemesis, abdominal pain, nausea, vomiting, diarrhoea, constipation or melena. On physical examination, the patient was hypotensive with a blood pressure of 89/65mmHg. She received adequate fluid resuscitation. Her abdominal exam was significant for multiple cutaneous neurofibromas but otherwise unremarkable. On rectal examination, she had yellow-coloured stools with no blood. Initial labs showed a significant decrease in haemoglobin (Hgb) from a baseline of 11.3g/dl to 7.9g/dl and an increased blood urea nitrogen to creatinine (BUN/Cr) ratio of 56. Other laboratory values were unremarkable. Esophagogastroduodenoscopy (EGD) revealed no signs of bleeding, and her colonoscopy revealed blood clots throughout the colon with no obvious colonic source contrary to the physical exam. On terminal ileum intubation, bright red blood was found. A video capsule endoscopy was arranged to identify the source of bleeding (figure 1).

**Figure 2:**
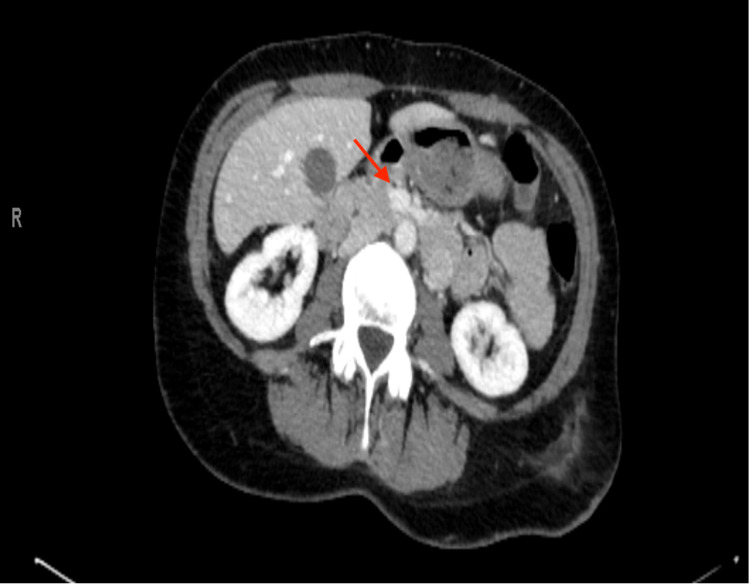
CT abdomen with IV contrast: A round partially exophytic avidly enhancing mass measuring 1.6x1.5cm in the 4th portion of the duodenum, favoured to represent a GIST. GIST - Gastrointestinal Stromal Tumor

**Figure 3:**
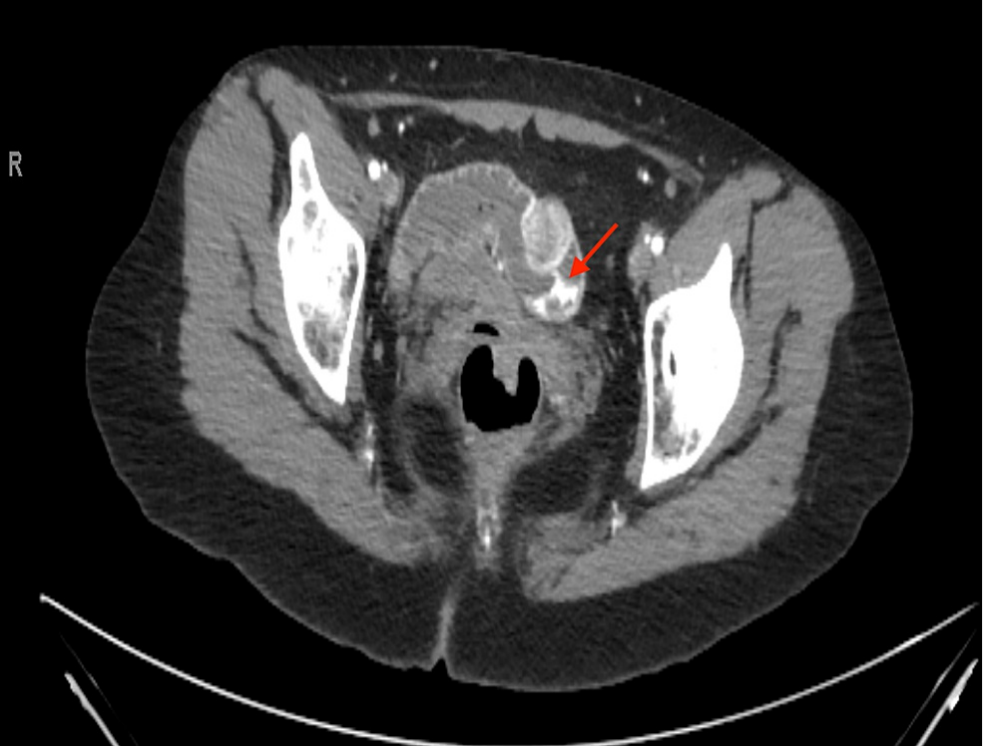
CT Abdomen with IV contrast: A lobulated endophytic avidly enhancing mass is identified in the Ileum, which measures approximately 2.4x2.2cm. This lesion demonstrates brisk active arterial bleeding into the intestinal lumen.

Given the history of NF-1 and ongoing severe GI bleeding, an urgent CT scan of the abdomen with contrast was obtained, which showed several avidly enhancing exophytic masses identified throughout the small bowel (figure 2). The largest of these lesions was in the anterior wall of the mid-ileum, measuring 2.4x2.2cm with active, brisk arterial bleeding into the intestinal lumen (figure 3).

**Figure 4:**
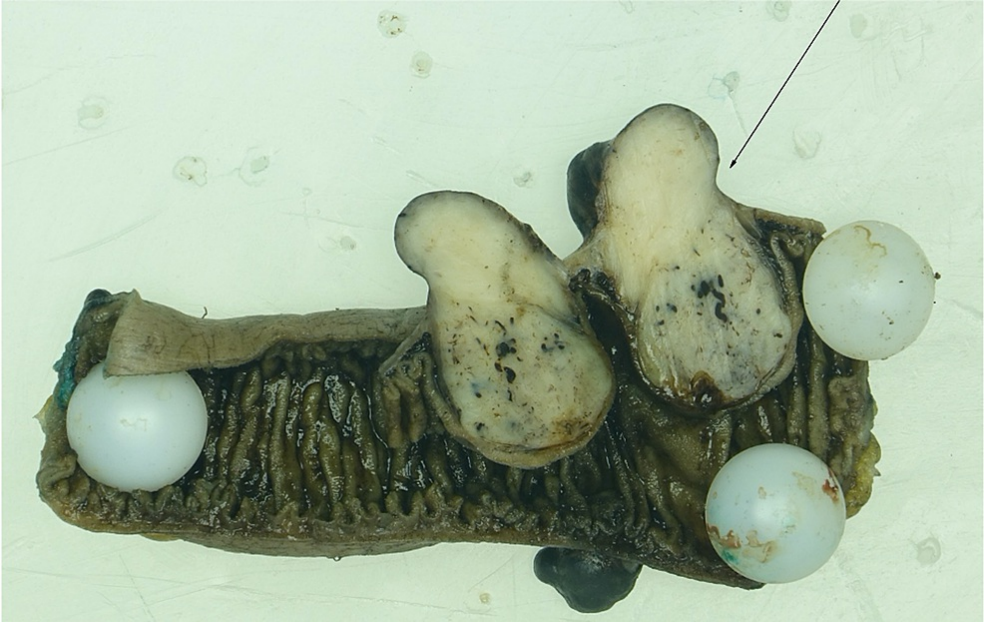
Opened segment of small bowel showing normal variegated mucosa with a cross section through the largest exophytic nodule, measuring approximately 2.2x1.8x1.6 cm.

**Figure 5:**
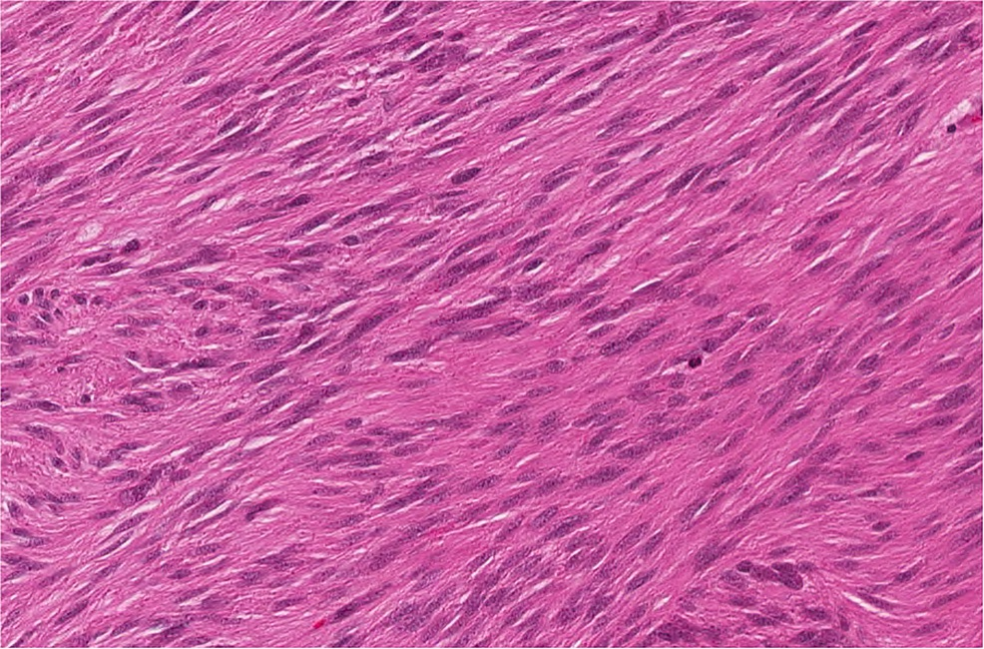
High power image of the lesion showing uniform, spindled cells with eosinophilic cytoplasm typical of a GIST. GIST - Gastrointestinal Stromal Tumors

An emergent surgical consult was placed, and the patient underwent surgical resection of the ileal segment containing an actively bleeding mass (figure 4, 5). Intraoperatively, there was a non-obstructive proximal jejunal mass distal to the Ligament of Treitz with several small bowel nodules measuring 1 cm or less. An exophytic mass measuring 2.2x1.8x1.6cm was noted near the proximal ileum that extended intraluminally and was likely the source of the bleed. Curative resection of this mass along with a 10-cm segment of adjacent bowel was performed. A side-to-side anastomosis was subsequently completed. There were no postoperative complications, and the patient did not have further episodes of melena or bleeding.

The histopathology of the resected ileal segment was positive for CD4, C-KIT & DOG-1, confirming GISTs. DOG-1 immunochemical staining is a sensitive and specific marker for GIST. The patient was started on Imatinib 400mg daily. Restaging with PET and CT scans revealed no evidence of new or progressive disease except for mildly increased activity within the mesenteric right lower quadrant lymph nodes measured less than 1cm. She remained symptom-free and tolerated Imatinib without adverse effects. The patient will be monitored with serial scans every six months.

## Discussion

NF is an autosomal dominant disease that presents with neuro-cutaneous lesions. It is subdivided into three classes: neurofibromatosis types 1, 2, and schwannomas. NF-1 is more frequently seen compared to the latter two. Cafe au lait spots and neurofibromas are commonly seen in cutaneous findings of this disease. GIST is seen in one-third of patients with NF-1. Only 5% of these patients are symptomatic [5]. GISTs are rare mesenchymal origin GI tumours constituting less than 1% of all GI tumours [5]. They usually present with abdominal pain, upper or lower GI bleeding, abdominal mass, fatigue, dysphagia, or early satiety [6].

Endoscopy is generally of low yield in diagnosing actively bleeding GISTs compared to CT angiography of the abdomen because of its inaccessible location in the small bowel. Although GISTs are more commonly located in the stomach, the incidence of bleeding is more frequently seen in non-gastric GIST [7-9]. GISTs associated with NF-1 are often numerous and located in the distal part of the small intestine [9]. In our case, GISTs were suspected due to the history of NF-1, which led to early diagnosis via imaging and timely management.

Anderson et al. showed that all NF-1 patients diagnosed with GIST had cafe-au-lait spots and/or histologically verified cutaneous and/or deep neurofibromas, indicating these physical exam findings as very helpful in raising clinical suspicion for GIST [5]. These unusual skin lesions should prompt one to look for underlying undiagnosed GIST. The other incentive to identify this association is that the management of GISTs is essentially curative. In contrast, sporadic GISTs are typically solitary and are found in the stomach [10]. The median age of onset for sporadic GISTs is 60-65 years old compared to the median age of onset of NF-1 associated GISTs, 49-years-old [11]. Sporadic GISTs involve mutations in KIT, a receptor tyrosine kinase encoded by protooncogene c-kit, and platelet-derived growth factor receptor-α (PDGFRA). NF1-associated GISTs, on the other hand, are associated with the activation of the RAS/RAF/MAP kinase signalling pathway through loss-of-function NF1 gene mutations [12].

Surgical resection is a commonly used treatment modality for bleeding GISTs, while other options include adjuvant and neo-adjuvant Imatinib, a tyrosine kinase inhibitor [6,13,14]. Benjamin et al. showed increased survival with surgical resection compared to chemotherapy alone [15]. Immunohistochemistry of GISTs is paramount in guiding treatment decisions [11]. Patients with NF-1 associated GISTs and mutations in KIT and PDGFRA have been shown to have a favourable response to Imatinib compared to patients without these mutations [8].

## Conclusions

Our case describes life-threatening GI bleeding from GISTs in a patient with NF-1. A third of NF-1 patients have GIST. In NF-1 patients presenting with GI bleeding, GISTs should be part of the differential. Clinicians must have a low threshold for urgent abdominal imaging if endoscopy does not detect the source of GI bleeding. Emergent surgery stands alone as a curative form of therapy for actively bleeding GISTs in patients with NF-1.
